# Supplementary material for: The Ability of Bumblebees Bombus terrestris (Hymenoptera: Apidae) to Detect Floral Humidity is Dependent Upon Environmental Humidity
Source: Environ Entomol. 2022 Jul 28;51(5):1010–9. doi: 10.1093/ee/nvac049 (PMC9585368; doi:10.1093/ee/nvac049)
Supplement: nvac049_suppl_Supplementary_Material [file nvac049_suppl_supplementary_material.pdf]

SUPPLEMENTARY FIGURES AND TABLE for “**The ability of bumblebees *Bombus terrestris* (Hymenoptera: Apidae) to detect floral humidity is dependent upon environmental humidity**” by Harrison & Rands

Table S1. Simple contrasts for rewarding flower type. For all estimates, SE = 0.213, and *p* values adjusted using the Tukey method for comparing a family of three estimates.

	ambient			low humidity			medium humidity			high humidity		
	estimate	t_{132}	*p*	estimate	t_{132}	*p*	estimate	t_{132}	*p*	estimate	t_{132}	*p*
control - dry	-0.952	-4.48	**<0.001**	-1.298	-6.10	**<0.001**	-1.583	-7.44	**<0.001**	-1.274	-5.99	**<0.001**
control - humid	-1.274	-5.99	**<0.001**	-1.060	-4.98	**<0.001**	-0.905	-4.25	**<0.001**	-0.190	-0.90	0.644
dry - humid	-0.321	-1.51	0.289	0.238	1.12	0.504	0.679	3.19	0.005	1.083	5.09	**<0.001**

Table S2. Simple contrasts for environmental humidity type. For all estimates, SE = 0.213, and *p* values adjusted using the Tukey method for comparing a family of four estimates.

	control			dry rewarding			humid rewarding		
	estimate	t_{132}	*p*	estimate	t_{132}	*p*	estimate	t_{132}	*p*
ambient – high	0.214	1.01	0.746	-0.107	-0.50	0.958	1.298	6.10	**<0.001**
ambient – low	-0.429	-2.01	0.188	-0.774	-3.64	**0.002**	-0.214	-1.01	0.746
ambient – medium	-0.310	-1.46	0.468	-0.941	-4.42	**<0.001**	0.060	0.28	0.992
high – low	-0.643	-3.02	**0.016**	-0.667	-3.13	**0.011**	-1.512	-7.11	**<0.001**
high – medium	-0.524	-2.46	0.071	-0.833	-3.92	**<0.001**	-1.238	-5.82	**<0.001**
low - medium	0.119	0.56	0.944	-0.167	-0.78	0.862	0.274	1.29	0.573

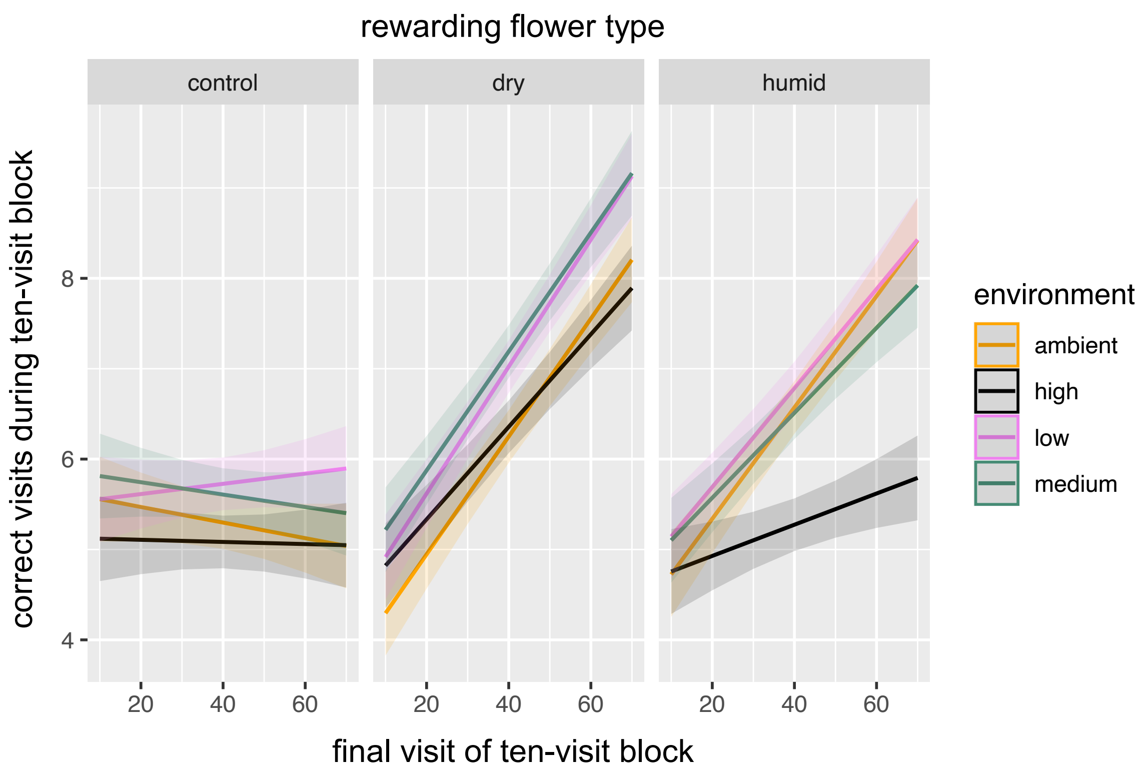

Figure S1. Interaction plots showing interactions for all three variables. Contrasts are detailed in Tables S1 and S2.
